# Transcriptomic-Guided Phosphonate Utilization Analysis Unveils Evidence of Clathrin-Mediated Endocytosis and Phospholipid Synthesis in the Model Diatom, *Phaeodactylum tricornutum*

**DOI:** 10.1128/msystems.00563-22

**Published:** 2022-11-01

**Authors:** Huilin Shu, Yanchun You, Hongwei Wang, Jingtian Wang, Ling Li, Jian Ma, Xin Lin

**Affiliations:** a State Key Laboratory of Marine Environmental Science, Xiamen University, Xiamen, China; b College of the Environment and Ecology, Xiamen University, Xiamen, China; c College of Ocean and Earth Sciences, Xiamen University, Xiamen, China; d National Observation and Research Station for the Taiwan Strait Marine Ecosystem, Xiamen University, Zhangzhou, China; e Xiamen Key Laboratory of Urban Sea Ecological Conservation and Restoration, Xiamen, China; University of California, Riverside

**Keywords:** phosphonate utilization, *Phaeodactylum tricornutum*, diatom, transcriptome, endocytosis, phospholipid

## Abstract

Phosphonates are important components of marine organic phosphorus, but their bioavailability and catabolism by eukaryotic phytoplankton remain enigmatic. Here, diatom Phaeodactylum tricornutum was used to investigate the bioavailability of phosphonates and describe the underlying molecular mechanism. The results showed that 2-aminoethylphosphonic acid (2-AEP) can be utilized as an alternative phosphorus source. Comparative transcriptomics revealed that the utilization of 2-AEP comprised 2 steps, including molecular uptake through clathrin-mediated endocytosis and incorporation into the membrane phospholipids in the form of diacylglyceryl-2-AEP (DAG-2-AEP). In the global ocean, we found the prevalence and dynamic expression pattern of key genes that are responsible for vesicle formation (*CLTC*, *AP-2*) and DAG-AEP synthesis (*PCYT2*, *EPT1*) in diatom assemblages. This study elucidates a distinctive mechanism of phosphonate utilization by diatoms, and discusses the ecological implications.

**IMPORTANCE** Phosphonates contribute ~25% of total dissolved organic phosphorus in the ocean, and are found to be important for marine phosphorus biogeochemical cycle. As a type of biogenic phosphonate produced by microorganisms, 2-aminoethylphosphonic acid (2-AEP) widely exists in the ocean. It is well known that 2-AEP can be cleaved and utilized by prokaryotes, but its ability to support the growth of eukaryotic phytoplankton remains unclear. Our research identified the bioavailability of 2-AEP for the diatom Phaeodactylum tricornutum, and proposed a distinctive metabolic pathway of 2-AEP utilization. Different from the enzymatic hydrolysis of phosphonates, the results suggested that P. tricornutum utilizes 2-AEP by incorporating it into phospholipid instead of cleaving the C-P bond. Moreover, the ubiquitous distribution of associated representative gene transcripts in the environmental assemblages and the higher gene transcript abundance in the cold regions were observed, which suggests the possible environmental adaption of 2-AEP utilization by diatoms.

## INTRODUCTION

Phosphorus (P) is an essential element for living organisms. It is involved in many cellular metabolic activities, such as the synthesis of nucleic acids and phospholipids of cell membranes. Dissolved inorganic phosphate (DIP) is the preferable form of P for microorganisms, but it is often scarce in the surface ocean (1). Dissolved organic phosphorus (DOP) is taken as the alternative P source under DIP deficiency, and the bioavailability of different DOP compounds has been widely examined (2). Marine microorganisms can utilize a broad spectrum of DOP compounds (C-O-P class) with diverse hydrolase enzymes, e.g., alkaline phosphatase activity (APA) is used as an indicator of P nutritional status (3–5). Phosphonates are a class of organophosphorus compounds containing C-P bond, which are estimated to contribute 25% of the total DOP (6) and play an important role in the P redox cycle (7). C-P bond is much more stable in comparison with C-O-P bond in phosphate esters, because it is resistant to chemical hydrolysis, thermal decomposition, and photolytic degradation (8).

Phosphonates are synthesized by a wide range of organisms and are mainly found as biogenic compounds either in free state or combined with proteins, lipids and, glycans in other organisms such as microorganisms, insects, and mammals (9–11). *Prochlorococcus* can synthesize phosphonates and incorporate them into cell-surface glycoproteins to protect cells from grazing and viral lysis (12). As such, 2-Aminoethylphosphonic acid (2-AEP) is the first identified natural phosphonate and one of the most abundant and ubiquitous phosphonates in the natural environment (8, 13), suggesting that it protects cells against predators while incorporated into membrane phospholipids (14). For example, glycerophospholipid DAG-2-AEP (8), a constituent in membrane phospholipids, is considered to protect the cells from enzymatic degradation or increase the structural rigidity attributed to the stability of C-P bond (15). Besides synthesis, phosphonate consumption genes are widely present in prokaryotic genomes in the global ocean, suggesting that oxidation and hydrolysis processes play an important role in the marine P cycle (12, 16, 17).

Biological and chemical evidence have proved that phosphonates can be an alternative P source for microorganisms (16). Although the utilization mostly occurs in phosphate-limited ocean regions such as the Mediterranean Sea and North Atlantic Ocean (16), 2-AEP can also be absorbed by prokaryotes when DIP is sufficient (17, 18). Catabolism of 2-AEP has been well elaborated in prokaryotes employing diverse pathways mediated by C-P lyase and C-P hydrolyses (19, 20). The C-P lyase pathway with broad substrate (e.g., 2-AEP and methylphosphonic acid) specificity, is more commonly present under P deficiency conditions in bacteria (20). Substrate-inducible C-P hydrolase pathway can hydrolyze phosphonates to release P, C, N, or energy sources (21). Transportation of extracellular 2-AEP into the cells through the phosphonate transporter complex is believed to be the precondition for these pathways, based on the previous finding that absence of transporters prevents C-P hydrolyses pathway from being functional in dinoflagellates (22). Previously identified 2-AEP transporters PhnCDE and PhnSTU both belong to ATP-binding cassette (ABC) transporter families, which are responsible for the transportation of multiple substrates (23, 24). Recently reported transporters AepXVW, AepP, and AepSTU show different affinities for 2-AEP (17).

Synthetic C-P compounds are widely used and have been detected in the coastal waters (25). Among them, the commonly used herbicides glyphosate (GLY) and glufosinate-ammonium (GLU) have been reported to support or inhibit cell growth dependent on different phytoplankton species (26, 27). To be more specific, diatoms Phaeodactylum tricornutum and Skeletonema costatum, and haptophytes Emiliania huxleyi and Isochrysis galbana can use GLY as the sole P source (26), while E. huxleyi and green algae Micromonas commode can use methylphosphonic acid, but only M. commode can utilize 2-AEP (28). Due to limited knowledge about the utilization of phosphonates in eukaryotic phytoplankton, how they are utilized remains to be answered. The most pressing research issues include: (i) the universality of the bioavailability of phosphonate compounds for eukaryotic phytoplankton, and (ii) the underlying mechanism of the assimilation or catabolism pathway.

Diatoms represent a major class of phytoplankton in the ocean, contributing ~45% of the primary production (29, 30). P. tricornutum is a model diatom species that has been extensively studied because of its ease of culture, different cell shapes, and ability to be genetically transformed (31, 32). Previous study reported that it can utilize synthetic phosphonate compound GLY (26). The completion of the whole genome sequence of P. tricornutum can provide fundamental data for further exploration of the cell metabolism (33). In this study, the bioavailability of biogenic phosphonate 2-AEP, together with synthetic phosphonates GLY and GLU for P. tricornutum was investigated. Then, comparative transcriptomic analysis was conducted to unveil the metabolic pathway regarding the utilization of extracellular 2-AEP by P. tricornutum. Based on the results, further exploration of environmental metatranscriptomics data analysis was performed to justify the proposed mechanism and discuss the ecological implications.

## RESULTS

### Promoted cell growth and physiological responses in 2-AEP culture.

**FIG 1:**
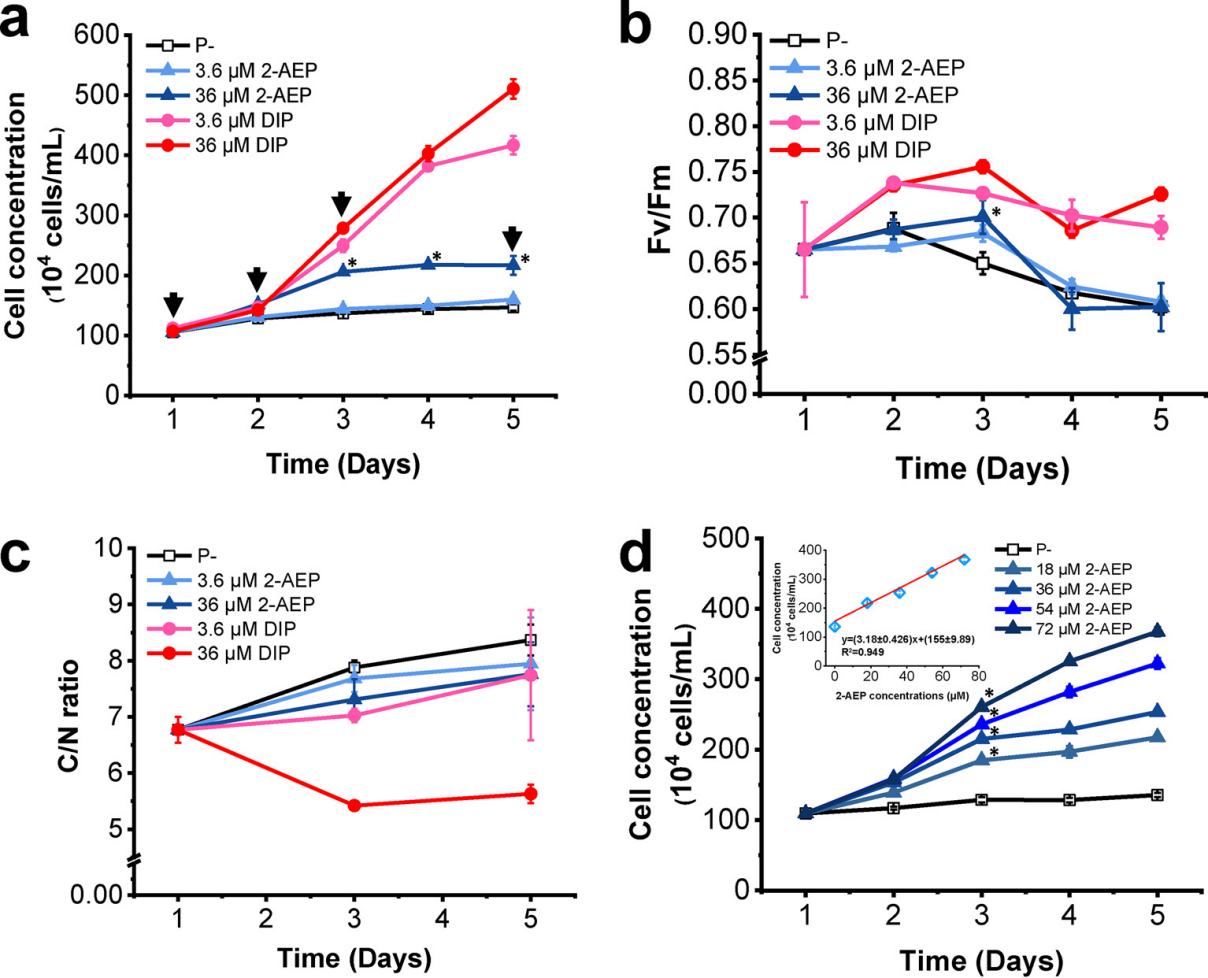
Physiological responses of P. tricornutum under different phosphorus conditions. (a) Growth curves, arrows denote the time to collect RNA samples. (b) Fv/Fm, (c) C/N ratio, (d) Cell concentrations grown under different 2-AEP concentrations, and correlation between 2-AEP concentrations and P. tricornutum cell density on D5 shown in up-left. Data are averages of biological triplicates; error bars show ± S.D. of biological triplicates. *P*-value was calculated by T-tests to represent significant difference between 2-AEP groups and P- group in (a), (b), and (d), *, *P* < 0.05.

In the batch-1 experiment, cell growth was arrested in the P-depleted group, and the cell density was one fifth of that of the DIP group (36 μM) on D5. In contrast, similar growth pattern and comparable cell density were observed in the 2-AEP group (36 μM) and the phosphonates mixture group (2-AEP+ GLY+ GLU, 36 μM each). This indicated that only 2-AEP can be utilized as the alternative P source by P. tricornutum, while GLY and GLU cannot be utilized (Fig. 1a and Fig. S1a). In comparison with the rapid growth in both DIP groups, cells exhibited weaker growth in cultures containing 36 μM 2-AEP and reached maximum cell density (216.9 ± 15.7 × 10^4^ cell/mL), about half of that was seen in the 36 μM DIP group (510.5 ± 16.0 × 10^4^ cell/mL) on D3, and then remained stable. In accordance with the cell growth, variable fluorescence/maximum fluorescence (Fv/Fm) (representing the photosynthetic capacity) exhibited a similar trend, i.e., DIP groups (3.6 and 36 μM) > 2-AEP groups (3.6 and 36 μM) > P− group (Fig. 1b and Fig. S1b). Significant increase (*P* < 0.05) in Fv/Fm value from 0.66 ± 0.05 to 0.73 ± 0.01 was observed in the DIP (36 μM) group (Fig. 1b), and no difference was identified between the P-depleted group and the other groups (Fig. S1b). In 2-AEP groups, Fv/Fm increased slightly, peaked on D3 (0.70 ± 0.02, close to the value of DIP 3.6 μM), and then decreased to the same level as the P− group (0.60 ± 0.01) (Fig. 1b). C/N ratio is an empirical indicator which is widely used in marine biogeochemistry studies, especially carbon export in the biological bump. It is reported that P deficiency leads to higher C/N ratio of phytoplankton, including P. tricornutum (34). Starting from C/N ratio ~6.8, the DIP (36 μM) group possessed the lowest value ~5.6, while the highest value ~8.4 was observed in the P− group, and cultures of both 2-AEP groups and DIP (3.6 μM) group shared the median value ~7.5 (Fig. 1c).

10.1128/msystems.00563-22.1 FIG S1 Physiological responses of *P. tricornutum* under different phosphorus conditions. (a) Growth curves, (b) Fv/Fm, (c) APA. (d) 2-AEP concentration in 2-AEP groups. Download FIG S1, TIF file, 1.5 MB. Copyright © 2022 Shu et al. 2022 Shu et al. https://creativecommons.org/licenses/by/4.0/ This content is distributed under the terms of the Creative Commons Attribution 4.0 International license.

### Linear relationship between cell density and 2-AEP concentration.

Briefly, physiological parameters showed that the utilization of 2-AEP was comparable to that of DIP (3.6 μM) by P. tricornutum, based on similar Fv/Fm and C/N ratio. Moreover, elevated APA (Fig. S1c) and arrested cell growth after D3 indicated that utilization was incomplete given that 2-AEP was provided in higher concentration. Thus, a gradient culture was set up to further explore the utilization of 2-AEP (Table 1). Results showed that stimulated cell growth was positively correlated with the increase in ambient 2-AEP concentration in a linear relationship (Fig. 1d). After 5 days, the highest cell concentration was observed in 72 μM 2-AEP group, about 4 times of that in the beginning, which is a stimulation comparable to that observed in 3.6 μM DIP (Fig. 1a and d). Moreover, concentrations of 2-AEP in the culture medium decreased both in 36 μM and 72 μM treatment groups, which indicated that 2-AEP was absorbed by P. tricornutum (Fig. S1d).

**TABLE 1:** Culture conditions in three culture batches

Culture	P nutrient concentration	T (°C)	Common condition
Seed	Starvation treated 8–10 day (<0.3 μM)	20	1. f/2 medium, salinity = 30 2. 14:10 light: dark cycle 3. photon flux: 180 μmol m^−2^ s^−1^ 4. Antibiotics cocktail (final concentration in medium: ampicillin 100 mg/L, streptomycin 50 mg/L and kanamycin 50 mg/L)
DIP supplied	DIP (3.6^*a*^, 36^*b*^ μM)		
DIP depleted^*c*^	No addition		
Batch 1	2-AEP^*d*^ (3.6, 36 μM separately) GLY^*e*^ (3.6, 36 μM separately) GLU^*f*^ (36 μM) 2-AEP+GLY+GLU (3.6 μM each) 2-AEP+GLY+GLU (36 μM each)		
Batch 2	2-AEP (18, 36, 54, 72 μM)		
Batch 3	2-AEP (72 μM)		

^*a, b, c*^control groups in Batch 1, ^*c*^control groups in Batch 2, ^*b, c*^control groups in Batch 3. ^*d*^2-AEP, ^*e*^GLY, and ^*f*^GLU standards were provided by Sigma-Aldrich (St. Louis, MO, USA).

### Overview of comparative transcriptomics.

**FIG 2:**
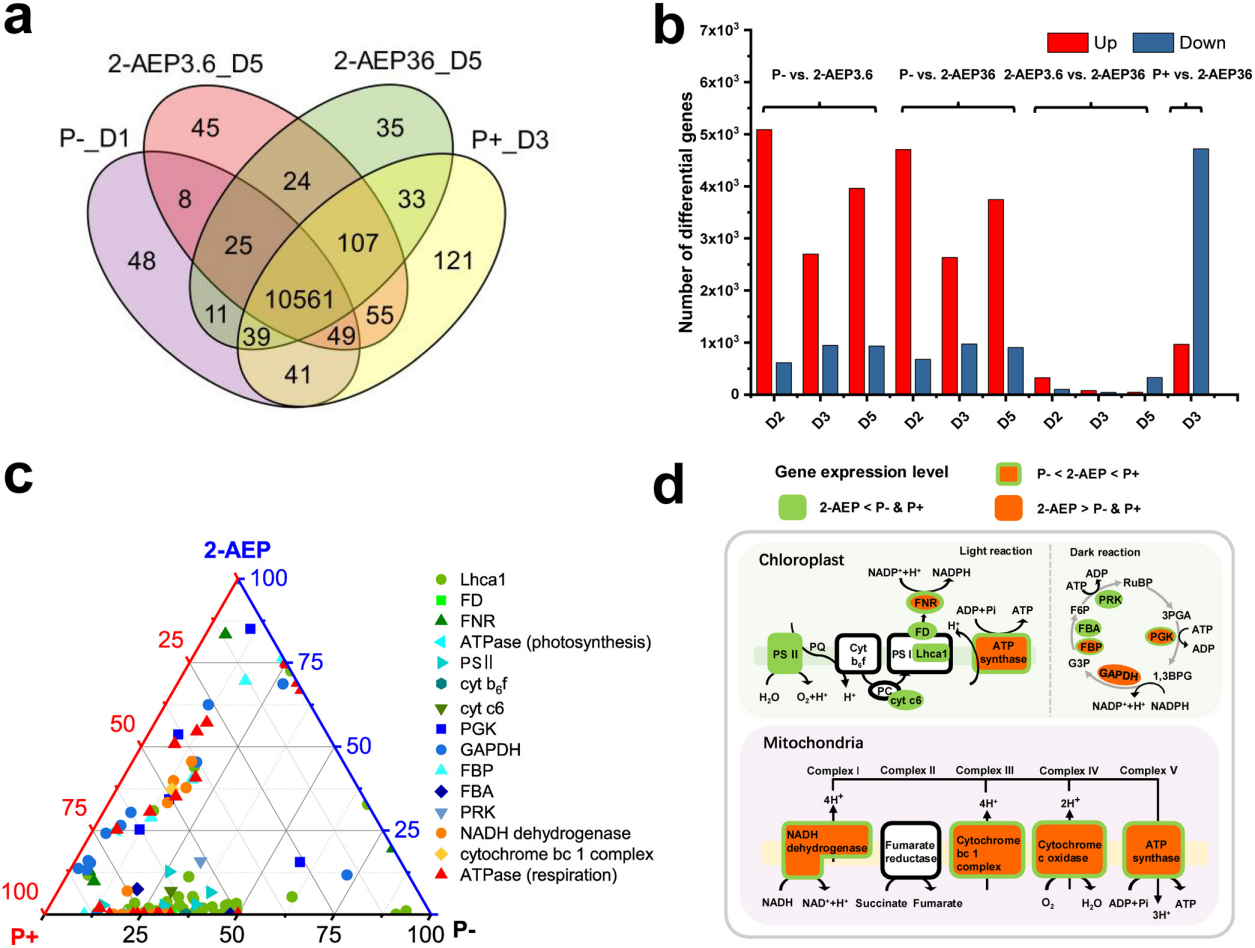
Comparative gene expression profiles of P. tricornutum under different phosphorus conditions. (a) Venn diagram showing shared genes among 2-AEP groups (3.6 μM and 36 μM, D5), P-depleted (P−, D1) group and P-replete (P+, D3) group. (b) Pairwise comparison of DEGs between different groups. (c) Ternary plots showing the DEGs of photosynthesis and respiration in P−, P+ and 2-AEP36_D5 groups. DEGs include LHCA1 (*LHCA1*), PS II (*psbO*, *psbU*, *psbQ*, *psbM*, *psb27*, *psbP*), FD (*petF*), FNR (*petH*), cyt b6f (*petC*), cyt c6 (*petJ*), ATP synthase in photosynthesis (*ATPF1A*, *ATPF1G*), PGK (*PGK*), GAPDH (*GAPDH*), FBP (*FBP*), FBA (*FBA*), PRK (*PRK*), NADH dehydrogenase (*NDUFA5*, *NDUFAB1*, *NDUFA2*, *NDUFS5*, *NDUFB7*, *NDUFB9*, *NDUFV2*, *NDUFA12*, *NDUFB10*, *NDUFS6*, *NDUFA9*), cytochrome bc_1_ complex (*QCR7*), ATP synthase in respiration (*ATPeV1H*, *PMA1*, *ATPF1A*, *ATP5O*, *ATP5D*, *ATP5A1*, *ATP5C1*, *ATP5E*, *ATP6C*, *ATPF1G*, *ATP6D*, *ATP6L*, *ATP6B*, *ATP6A*, *ATP6M*, *ATP6S14*). Each type of points corresponds to DEGs of components involved in photosynthesis and respiration. Its position represents its expression level with respect to each group (P−, P+, 2-AEP36_D5). (d) Schematic summary of metabolic activity differences in photosynthesis and respiration in P. tricornutum under different phosphorus conditions. Specific information of DEGs in different groups refers to Table S1c.

Raw reads were filtered to remove low quality reads (bases with quality values below 15 accounted for more than 20% of the total base of the reads), contaminating sequences, and reads containing unknown N bases higher than 5%. High-quality reads were assembled and mapped to the reference genome with the average mapping rate of 86.61% (Table S1a). A total of 11,293 genes was detected, including 11267 known genes and 26 unknown genes. Independent comparison between two 2-AEP groups (3.6 and 36 μM) revealed that most detected genes were shared by both (Fig. S2a and b). Samples of D5 of both 2-AEP groups (3.6 and 36 μM) were subjected to further comparative analysis against P− and P+ groups showing 10561 genes shared among 4 groups (Fig. 2a).

10.1128/msystems.00563-22.2 FIG S2 (a) Venn diagram of DEGs in 3.6 μM 2-AEP group on D2, 3, 5. (b) Venn diagram of DEGs in 36 μM 2-AEP group on D2, 3, 5. (c) GO classification of DEGs, red boxes indicate the most enriched term (function of membrane 23.8%, catalytic activity 13.3%). (d) KEGG classification of DEGs. (e) Comparison of log2 fold change between data obtained by RNA sequencing and PR-qPCR. (f) Log2 fold changes of selected genes involved in endocytosis and phospholipid synthesis in group P+ vs. 2-AEP36_D5, *ARFGAP* (*SMAP*), *HSP70* (*HSPA1s*). Download FIG S2, TIF file, 2.3 MB. Copyright © 2022 Shu et al. 2022 Shu et al. https://creativecommons.org/licenses/by/4.0/ This content is distributed under the terms of the Creative Commons Attribution 4.0 International license.

10.1128/msystems.00563-22.5 TABLE S1 (a) RNA-seq samples and reads mapping rate. Cells of group P− (D1, representing P starvation condition) and cells of group P + (D3, representing exponential stage in 36 μM DIP group) were used to conduct comparative transcriptomic analysis against 2-AEP groups (3.6 μM & 36 μM). (b) Numbers of DEGs in pairwise comparison between different groups. (c) FPKM of DEGs in different pairwise comparison groups. Download Table S1, XLSX file, 0.04 MB. Copyright © 2022 Shu et al. 2022 Shu et al. https://creativecommons.org/licenses/by/4.0/ This content is distributed under the terms of the Creative Commons Attribution 4.0 International license.

Through pairwise comparison among different groups (Fig. 2b and Table S1b), significantly higher numbers of differentially expressed genes (DEGs) were identified between P− versus 2-AEP (3.6 and 36 μM) and P+ versus 2-AEP (36 μM) group, but limited DEGs were identified compared to 2-AEP groups (3.6 μM versus 36 μM). Most of the DEGs were upregulated (1.00 ~ 11.93 Log2FC) in 2-AEP cultures compared with P− group, while most DEGs were identified as downregulated (1.00 ~ 12.75 Log2FC) compared with P+ group (Fig. 2b). Gene Ontology (GO) categorization showed that detected DEGs (P− versus 2-AEP36_D5) were significantly enriched in cellular process, metabolic process, membrane, binding, and catalytic activity (Fig. S2c). Regarding KEGG pathways mapping, most DEGs were enriched in the pathway related to metabolism, including lipid metabolism, energy metabolism, and global and overview maps (Fig. S2d).

### Differences in metabolic activity.

In general, comparative analysis of photosystem transcriptional response revealed that cells in 2-AEP groups had moderate gene expression levels compared to both P− and P+ groups (Fig. 2c and d, and Table S1c), which is consistent with observed Fv/Fm values (Fig. 1b). The light reaction of photosynthesis is composed of photosystem (PS II and PS I), electron transport chain (ETC) and generation of ATP (35). Transcriptional expression of genes involved in PS II (*psbO*, *psbU*, *psbP*, *psbQ*, *psbM*, *psb27*), PS I (*LHCA1*) and ETC [FD (*petF*)] was repressed in 2-AEP groups against P− and P+ groups (Fig. 2c and d, and Table S1c). Gene expression levels of *FNR* (*petH*) and *ATPase* (*ATPF1A*, *ATPF1G*) of 2-AEP groups were situated between P− and P+ groups (Fig. 2c and d, and Table S1c). In the dark reaction where carbon fixation occurs, *PGK* and *GAPDH* involved with reduction were upregulated compared to the P− group, whereas the genes *FBA* and *PRK* involved with RuBP regeneration were downregulated compared to both P− and P+ groups (Fig. 2c and d, and Table S1c). Gene transcripts associated with oxidative phosphorylation of mitochondria (NADH dehydrogenase, cytochrome bc 1 complex, cytochrome c oxidase, and ATP synthase) exhibited similar expression pattern, and were situated between the P− and P+ groups (Fig. 2c and d, and Table S1c).

Furthermore, comparable upregulation of P-stress marker genes was identified in 2-AEP groups and P− group in comparison with P+ group (Table S2). In accordance with elevated APA in the culture, AP genes *PhoA* (Phatr3_J47869) and *PhoD* (Phatr3_J45959) were upregulated by 4.45 and 2.49 Log2FC, respectively (P+ versus P−), and by 6.39 and 2.57 Log2FC (P+ versus 2-AEP36_D3), similar to the reported values of 6.84 and 2.09 Log2FC (36). Phosphate transporter genes *NPT* (Phatr3_J40433, Phatr3_J47667) exhibited values of 1.51 and 2.95 Log2FC (P+ versus P−), and 1.98 and 2.92 (P+ versus 2-AEP36_D3), also similar to the previous study (36). The above results indicated that cells grown in P-depleted 2-AEP group exhibited a metabolic reconfiguration despite being under P-stress. Guided by these findings, further efforts were made to decipher the underlying molecular mechanism involved in 2-AEP utilization by comparing 2-AEP groups against the P-depleted group.

10.1128/msystems.00563-22.6 TABLE S2 Comparison of P-stress gene markers between this study and previous reports. Download Table S2, DOCX file, 0.01 MB. Copyright © 2022 Shu et al. 2022 Shu et al. https://creativecommons.org/licenses/by/4.0/ This content is distributed under the terms of the Creative Commons Attribution 4.0 International license.

### Uptake of 2-AEP through clathrin-mediated endocytosis.

Barely detectable DIP (lower than the detection limit ~0.3 μM) in 2-AEP culture suggested that 2-AEP might be transported into the cells to be utilized. However, gene coding phosphonate transporters PhnCDE, PhnSTU, AepXVW, AepP, and AepSTU reported previously (17) were not identified in all examined groups (absence in the transcriptome). Instead, comprehensive analysis revealed that 2-AEP was transported into cells encapsulated by the clathrin-coated vesicles.

**FIG 3:**
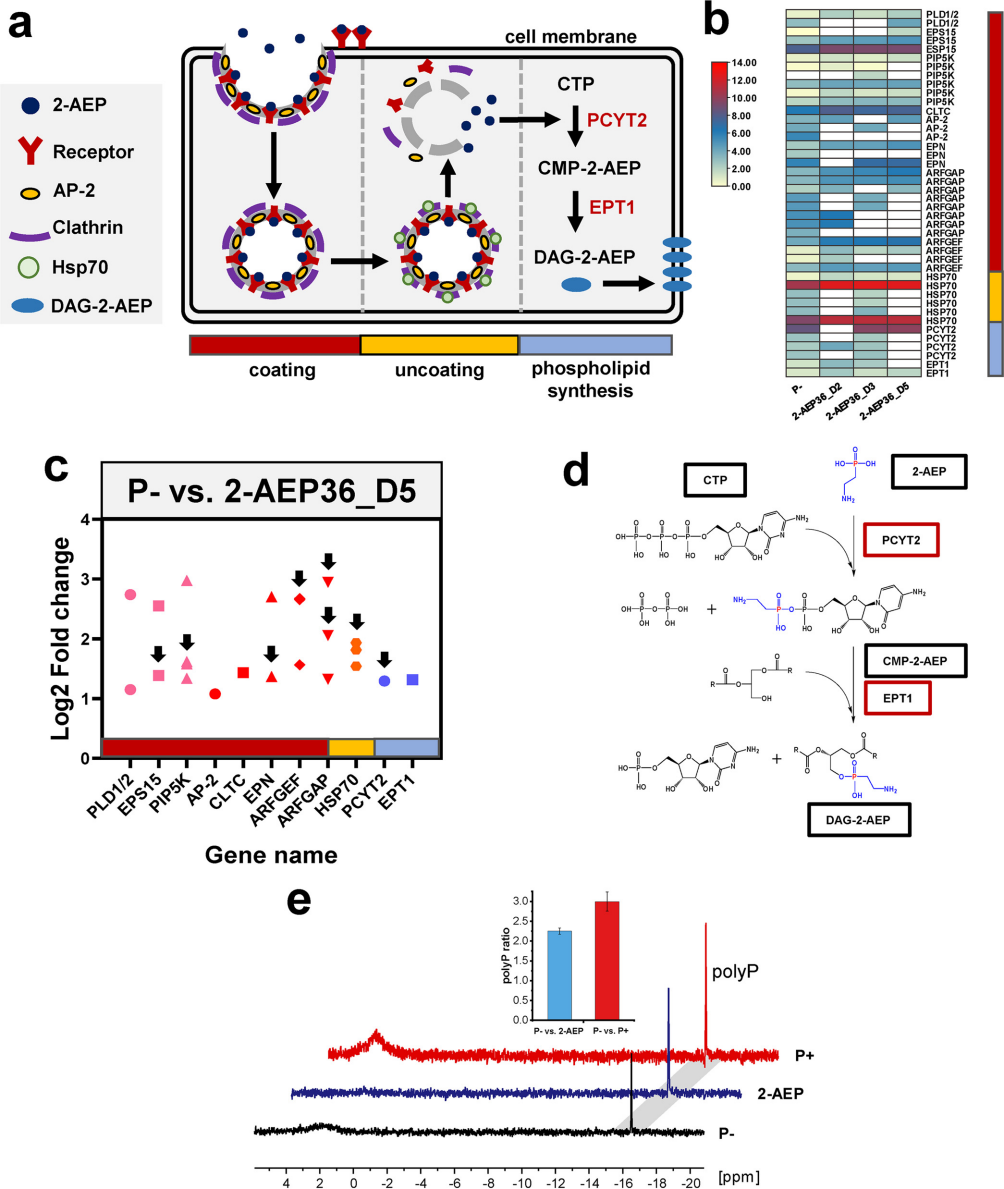
Metabolic pathway of 2-AEP utilization by P. tricornutum. (a) Schematic summary of metabolic pathway of 2-AEP utilization by P. tricornutum. Coating process, 2-AEP encapsulated by the clathrin-coated vesicles, receptor genes including *EPS15*, *PLD1/2*, *PIP5K*. Uncoating process, 2-AEP released from the vesicles driven by HSP70 binding. Phospholipid synthesis, incorporation of 2-AEP into cell membrane catalyzed by two enzymes, ethanolamine-phosphate cytidylyltransferase (PCYT2) and ethanolaminephosphotransferase (EPT1). CMP-2-aminoethylphosphonate (CMP-2-AEP), Diacylglyceryl-2-aminoethylphosphonate (DAG-2-AEP). (b) Expression level (normalized FPKM values) of multi-copy genes in time series samples of 2-AEP36, P- represent the initial cell condition, color bar represents the normalized gene expression level. *ARFGAP* (*SMAP*, *AERGAP1*), *ARFGEF* (*ARFGEF*, *GBF1*), *HSP70* (*HSPA1s*). (c) Fold changes of selected genes involved in endocytosis and phospholipid synthesis in group P− versus 2-AEP36_D5, genes *EPS15*, *CLTC*, *HSPA1s*, *PCYT2* and *EPT1* were verified by RT-qPCR (Fig. S4a), *ARFGAP* (*SMAP*), *HSP70* (*HSPA1s*). Arrows represent genes upregulated in P+ versus 2-AEP group as well. (d) Phospholipids biosynthesis pathway showing the incorporation of 2-AEP. Black boxes represent compounds, red boxes represent enzymes. (e) ^31^P-NMR spectra under different phosphorus conditions. The histogram (Inset) displays the PolyP ratios of P− versus 2-AEP (blue) and P− versus P+ (red).

Clathrin is a self-assembling protein that can form cage-like lattices at the plasma membrane to perform vesicular uptake (37). Clathrin-mediated endocytosis is a classical manner adopted by eukaryotes to transport cargo molecules into cells, characterized by a well-orchestrated process including coating (activation, vesicle formation) and uncoating. Up-regulation of gene *CLTC* was identified, coding clathrin heavy chain with intact conserved domains, clathrin-link, and 7 alpha helical repeats region (Fig. S3a and b). Besides this key gene, the upregulation of a whole train of genes involved with endocytic process was observed in 2-AEP groups compared with the P− group (Fig. 3a, b, and c, and Fig. S2e).

**FIG 4:**
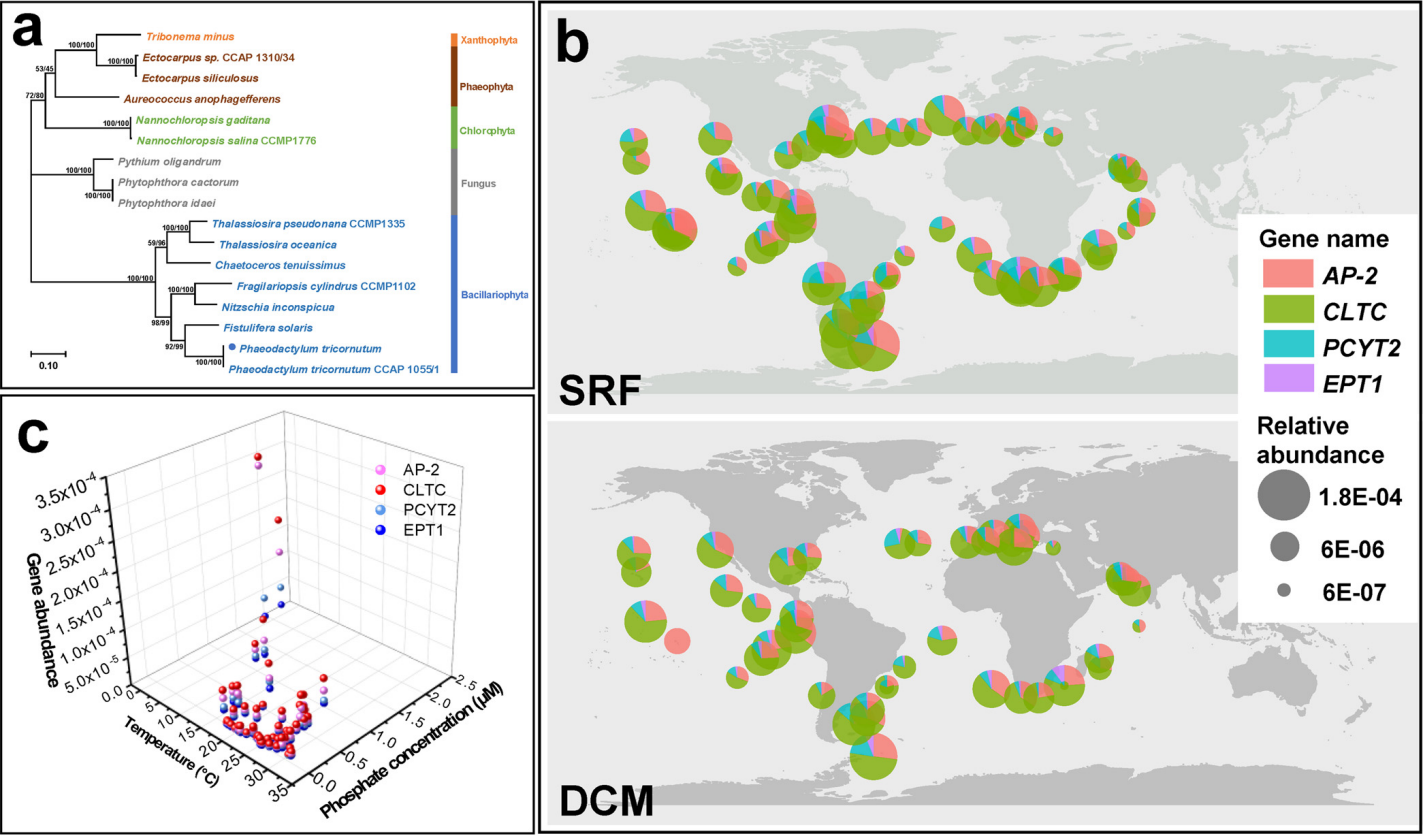
(a) Phylogenetic tree inferred from amino acid sequences of clathrin of P. tricornutum and other eukaryotic organisms (Sequence IDs listed in Table S4a). Tree topology shown as generated from ML. Support nodes obtained from ML/NJ are shown as percentage. The blue dot represents the species used in this study. (b) Biogeographic distribution of gene *AP-2*, *CLTC*, *PCYT2* and *EPT1* of diatom in the ocean including the layer of surface (SRF) and the deep chlorophyll maximum (DCM). (c) Distribution of selected genes involved in 2-AEP utilization with respect to environment parameters (temperature and phosphate concentration) in surface waters (5 m depth).

10.1128/msystems.00563-22.3 FIG S3 Alignment of deduced CLTC, PCYT2, and EPT1 sequences in different species. (a) A full length CLTC amino acid sequence with conserved domains. The Purple box represents clathrin-link domain, and the green boxes represent helix region in the arm of the clathrin heavy chain (b) CLTC protein sequence alignment of *P. tricornutum* and several related species which used to construct the phylogenetic tree in Fig. 4a. The red box represents clathrin-link domain. The green box represents alpha helix region in the arm of clathrin heavy chain (The other 6 alpha helix region were not shown). (c) The sequence with conserved catalytic domain CTP_transfer_like. (d) The red box represents the catalytic domain of PCYT2 sequences which used to construct phylogenetic tree in Fig. S4b. (e) A full length EPT1 amino acid sequence with conserved domains CDP-OH_P_transfer. (f) The red box represents the catalytic domain of EPT1 sequences which used to construct the phylogenetic tree in Fig. S4c. Download FIG S3, TIF file, 2.5 MB. Copyright © 2022 Shu et al. 2022 Shu et al. https://creativecommons.org/licenses/by/4.0/ This content is distributed under the terms of the Creative Commons Attribution 4.0 International license.

10.1128/msystems.00563-22.4 FIG S4 Phylogenetic trees inferred from amino acid sequences of AP-2, PCYT2, EPT1 of *P. tricornutum* and other eukaryotic organisms (Sequence IDs listed in Table S4a). (a) AP-2, (b) PCYT2, (c) EPT1. Tree topology shown as generated from ML. Support nodes obtained from ML/NJ are shown as percentages. The blue dot represents the species used in this study. Download FIG S4, TIF file, 4.1 MB. Copyright © 2022 Shu et al. 2022 Shu et al. https://creativecommons.org/licenses/by/4.0/ This content is distributed under the terms of the Creative Commons Attribution 4.0 International license.

First, membrane coating process, including activation and vesicle formation, was accomplished through a complex protein interaction network. *EPS15*, *PLD*, and *PIP5K* are receptor genes coding for the protein responsible for endocytosis initiation, cargo recruitment, and signal transduction, respectively (38–40). Genes *PLD1/2*, *EPS15*, and *PIP5K* exhibited values of 1.1~3.5 Log2FC in both 2-AEP groups (D2, 3, 5) compared with P-depleted group (Fig. 3b and c, and Table S1c). According to the expression level of these multi-copy genes in time series samples of the 2-AEP36 group, most genes reached their highest transcriptional level on D5, and selected genes were upregulated within the range of 1.0~3.0 Log2FC (Fig. 3b and c). After activation, *AP-2*, *clathrin* (*CLTC*), and *EPN* genes were activated to form endocytic cups, which were upregulated within the range of 1.0~2.7 Log2FC in all 2-AEP samples compared with the P− group (Fig. 3a and c, and Table S1c) (41–43). Different from those genes, *ARFGEF* and *ARFGAP* (*SMAP*) genes playing important roles in membrane trafficking and actin remodeling (44, 45) were upregulated mostly on D2, and *ARFGEF* maintained higher expression till D5 with 1.6~2.7 Log2FC (Fig. 3b and c, and Table S1c).

Regarding the uncoating process, heat shock protein HSP70 is responsible for binding to clathrin and driving the dissociation of coated vesicles (46). Results showed that *HSP70* (*HSPA1s*) gene expression was significantly upregulated (1.5~2.7 Log2FC) in both 2-AEP groups (D2, 3, 5) compared to the P− group (Fig. 3a, b, and c, and Table S1c). The upregulation of selected genes *EPS15* (Phatr3_J42442), *CLTC* (Phatr3_EG01984), and *HSPA1s* (Phatr3_J54019) was further verified by RT-qPCR in the case of P− versus 2-AEP36_D5 (Fig. S2e and Table S3). In addition, the upregulation of selected genes (indicated by arrows in Fig. 3c) was also identified compared with the P+ group (Fig. S2f and Table S1c).

10.1128/msystems.00563-22.7 TABLE S3 Primers used in RT-qPCR. Download Table S3, DOCX file, 0.01 MB. Copyright © 2022 Shu et al. 2022 Shu et al. https://creativecommons.org/licenses/by/4.0/ This content is distributed under the terms of the Creative Commons Attribution 4.0 International license.

### Incorporation of 2-AEP into phospholipids.

It was proposed that after, endocytosis into P. tricornutum cells, 2-AEP was incorporated into the phospholipids under catalysis (Fig. 3a and d). According to the KEGG pathway (ko00440), 2-AEP first reacted with CTP catalyzed by ethanolamine-phosphate cytidylyltransferase (PCYT2) to produce CMP-2-aminoethylphosphonate (CMP-2-AEP) and diphosphate. Subsequently, CMP-2-AEP and 1,2-diacyl-sn-glycerol were catalyzed by ethanolaminephospho-transferase (EPT1) to form diacylglyceryl-2-AEP (DAG-2-AEP), a component of membrane phospholipids (Fig. 3d). The catalytic domain of PCYT2 and EPT1 was identified (Fig. S3c and d, and Fig. S3e and f). These 2 key enzymes catalyzing the biosynthesis of DAG-2-AEP were significantly upregulated in 2-AEP36 group (1.3~4.5 Log2FC) compared with the P− group and P+ group on D5 (Fig. 3b and c, and Fig. S2f, and Table S1c). The differential expression of genes *PCYT2* (Phatr3_J40163) and *EPT1* (Phatr3_J37086) in P− versus 2-AEP36_D5 group was verified by RT-qPCR as well (Fig. S2e).

### ^31^P nuclear magnetic resonance spectra.

^31^P nuclear magnetic resonance (NMR) spectroscopy was employed to investigate the P-containing compounds present in the cells under different P conditions. Surprisingly, a single independent signal at −16.5δ was observed in the ^31^P NMR spectrum (Fig. 3e), representing the abundant presence of polyphosphate (PolyP) (47). After normalization by cell numbers, it was found that the cellular PolyP content in both 2-AEP and DIP groups was more than twice as much as that in the P− group (Fig. 3e).

### Prevalence and transcript abundance of selected genes in environmental samples.

Genes *CLTC*, *AP-2*, *PCYT2*, and *EPT1*, representing endocytosis and incorporation process of 2-AEP utilization by diatom, were selected for further analysis. The selected genes were found to be widespread in the reported diatom genomes, including P. tricornutum, Fistulifera solaris, Nitzschia inconspicua, Fragilariopsis cylindrus, Chaetoceros tenuissimus, Thalassiosira pseudonana and Thalassiosira oceanica. Further phylogenetic analysis of deduced amino acid sequences derived from diatom and other representative eukaryotic organisms showed that selected genes of diatom formed an independent branch in the phylogenetic tree (Fig. 4a, Fig. S4, and Table S4a).

10.1128/msystems.00563-22.8 TABLE S4 (a) Sequence ID of species selected in phylogenetic tree (* The sequence we submitted to NCBI). (b) Data retrieved from Ocean Gene Atlas used in Fig. 4b. (c) Gene annotation and abundance retrieved from Southern Ocean diatom assemblages. Download Table S4, XLSX file, 0.07 MB. Copyright © 2022 Shu et al. 2022 Shu et al. https://creativecommons.org/licenses/by/4.0/ This content is distributed under the terms of the Creative Commons Attribution 4.0 International license.

Based on this result, a further exploration of the biogeographic distribution of these selected gene transcripts involved with the above-described mechanism was conducted using the environmental meta-omics data set, Ocean Gene Atlas (16, 48). A ubiquitous distribution of these gene transcripts was found in diatom assemblage worldwide, in both surface and DCM water layers (Fig. 4b). In general, transcripts of selected genes were actively expressed globally, showing similar biogeographic distribution patterns. Specifically, relatively lower abundances were detected in the DCM layer compared to the surface layer (Fig. 4b and Table S4b). Plotting abundances of selected gene transcripts from surface samples against in-situ temperature and P concentration showed significantly enriched distribution (85% of total stations) of selected gene transcripts in waters with mild temperatures ranging from 15 to 30°C and low phosphate concentration (0 to 0.5 μM) (Fig. 4c and Table S4b). Furthermore, it was observed that elevated abundances of individual samples moved toward colder water (<10°C) with higher phosphate concentration (>1 μM). Samples with highest transcript abundance of all selected gene transcripts were collected at the Southern Ocean (62.14°S, 49.3273°W) with the highest phosphate concentration (~2 μM) and lowest temperature (~0°C) (Fig. 4c and Table S4b). By searching through the Southern Ocean (SO) diatom assemblage transcriptome database (49), abundant transcript expressions of four selected genes were also identified (Table S4c) in diatom species shown in Fig. 4a.

## DISCUSSION

### Cargo transportation through clathrin-mediated endocytosis.

Clathrin-mediated endocytosis transports different kinds of extracellular molecules into the cells through vesicular trafficking (50). This process, requiring different components to work together to drive the formation and dissociation of vesicles, has been elucidated in many eukaryotic model organisms sharing similar modules as it is, for example, the major endocytic route in plants and is well elaborated in the root iron uptake (51). Yet, until now, there are limited reports describing this mechanism in phytoplankton, in which uptake of nutrients from the environment is crucial for their growth and primary production in the ocean. Endocytosis mechanism has been proposed in the silicon capture in diatoms, but the experimental evidence is lacking (52). In P. tricornutum, the comparative experiments between mutant and wild type cells evidenced that Fe acquisition was realized through endocytosis-mediated siderophore uptake (53). In E. huxleyi, P starvation enhanced formation of membrane vesicles in the cytoplasm (54). Besides, in dinoflagellate P. donghaiense and green algae Chlamydomonas reinhardtii, this process has been reported to be a route for both nutrients and harmful substances to enter the algal cells, respectively (55, 56). However, the metabolic pathway of this mechanism and dynamic expression under different nutrient conditions were not elaborated before.

Clathrin-mediated endocytosis is considered to be a stochastic process (57). The fundamental principle of cargo recruitment is that the concentrated transmembrane cargo molecules bind with the clathrin protein coat at the region of the plasma membrane, then form the vesicle, and are endocytosed (58). Substances with higher concentrations result in concentrated cargo molecules, which then have a greater opportunity to combine with the adaptors of the clathrin protein coat components to increase the likelihood of endocytosis initiation (57). In this study, the positive correlation observed between the stimulated cell growth and elevated 2-AEP concentration in ambient conditions (Fig. 1d) is consistent with the above-described characteristics of clathrin-mediated endocytosis. Plants use root iron transporter IRT1 to uptake ambient Fe, which is internalized from the plasma membrane through clathrin-mediated endocytosis (51). In the iron uptake study of P. tricornutum, the key protein ISIP1 containing an AP2-recognizing motif plays an important role in the endocytosis process, suggesting clathrin-mediated endocytosis (53). Taking these together, it is possible that there is a mediator protein (cargo molecules) required to bind with phosphonate to trigger endocytosis, which requires further exploration.

The worldwide distribution and enriched expression of key endocytosis genes in low phosphate regions suggest that this mechanism might be a prevalent strategy employed by diatoms in the pelagic environment. Characterized by randomness, it is reasonable to deduce that diverse forms of nutrients can be adopted through the vesicle mechanism as well.

### Incorporation of 2-AEP in membrane phospholipid and physiological implications.

Phospholipids and nucleic acids are the major P sources for phytoplankton cell growth (59). Phospholipids are the main constituents of cell membranes, and glycerophospholipids account for the vast majority of phospholipids (60). Glycerophospholipids are especially important for structural and functional components of the cell membrane (61). It was reported that 2-AEP first reacts with CTP to form CMP-2-AEP, which is then transferred to diglyceride to form the glycerophospholipid (8). The process of DAG-2-AEP synthesis has been described in animal tissues (62, 63). Though there is no report on the presence of DAG-2-AEP in algae cells, the biosynthesis pathway can be depicted based on the transcriptomic data obtained in this study. Based on the upregulation of 2 enzymes that catalyze the incorporation of DAG-2-AEP from 2-AEP, it can be hypothesized that P. tricornutum uses 2-AEP to synthesize cell membrane lipids to maintain the cell structure and morphology. This allows the cells to reallocate the P distribution among cellular metabolisms under P-stress condition. Elevated 2-AEP levels and redistribution of lipids were identified in the fusion site during Tetrahymena mating, suggesting the function of 2-AEP in membrane bending (64). In addition, our results showed that the cellular C/N ratio of P. tricornutum was strongly affected by P nutrient condition, and the lowest value of 5.6 (P+) was consistent with the value of 5.64 reported for P. tricornutum under P sufficient conditions (65). Median C/N ratio of 2-AEP group compared with the P-depleted group also indicated that cells were partially relieved from P-stress and managed to adjust the cellular resource allocation.

Impeded photosynthesis but elevated respiration was observed in 2-AEP group, suggesting a higher demand in energy (Fig. 1b, and Fig. 2c and d), which may be consumed by the endocytosis and membrane incorporation processes. Lower cell density and photosystem gene transcript expression indicated that 2-AEP is an alternative P source compared with the preferred DIP for P. tricornutum.

According to many previous studies, bacteria and cyanobacteria can utilize 2-AEP by cleaving the C-P bond of 2-AEP to form phosphate (18). In contrast, the 2-AEP utilization mechanism of eukaryotic P. tricornutum unveiled in this study is disparate. Further, 2-AEP is chemically stable and its dissociation energy is much higher than that of other types of organic P (17, 66). Regarding energy consumption, P. tricornutum cells might save energy through direct incorporation of 2-AEP into the phospholipids instead of cleaving the C-P bond to retrieve the phosphate, which would be a cost-effective approach under phosphate-limited conditions. There have been many reports regarding lipid remodeling and phospholipid decrease under P deficiency conditions in different phytoplankton species (67). However, instead of phospholipid, different cellular PolyP contents were identified by ^31^P NMR herein. PolyP is considered as the luxury P storage, playing an important role in the P deficiency condition (36). Therefore, to manage intracellular P allocation for cell metabolism, P. tricornutum of the 2-AEP group were able to reduce the hydrolysis of PolyP while lipid requirements were met by 2-AEP supply.

It was observed that P. tricornutum could utilize 2-AEP but not GLY, which is different from reported studies (26, 28). Considering this discrepancy, the present results can be explained from 2 aspects. Regarding the utilization of 2-AEP, the seed culture used in this study was subjected to P starvation, which could enhance the possibility of absorbing diverse DOP compounds, as discussed in Karl, 2007 (68). Differential cell growth response to GLY was observed in a previous report (26). After antibiotics were provided in the medium, GLY was found to inhibit the cell growth instead of slight promotion observed in the medium without antibiotics (26). A follow-up report also showed that the bacterial community might play a key role in the hydrolysis of GLY to support cell growth in dinoflagellate (69). In comparison, a consistent growth pattern was observed in both single compound culture and mixed compound culture with antibiotics provided, suggesting a solid result.

Furthermore, at the community level, higher relative abundances of selected genes in cold waters and enriched distribution in low P waters were identified. Thus, it can be speculated that the utilization of 2-AEP can be adopted as a survival or competitive strategy for diatoms under P deficiency or low temperature. At present, P deficiency in the ocean is prevalent, and the DOP concentrations in the pelagic ocean are higher than DIP concentrations in the surface water (5). These results showed relative higher expression of selected genes in surface samples, which is consistent with this pattern. Diatom plays an important role in carbon fixation. The phosphonate utilization by diatoms is crucial for primary production under P deficiency. Under these circumstances, the phosphonate bioavailability and utilization exemplified by diatoms should be further examined to evaluate the contribution to biomass, primary production, and the biogeochemical cycle of dissolved and particulate P pools in the ocean.

### Conclusions.

This study showed that biogenic phosphonate 2-AEP can promote the growth of P. tricornutum under P deficiency. Through comparative omics analysis, an unconventional mechanism adopted by P. tricornutum to utilize extracellular 2-AEP was elaborated. It was proposed that 2-AEP is transported into the cells through clathrin-mediated endocytosis and then incorporated into the membrane phospholipids. Selected genes representing this deductive mechanism are actively expressed throughout the ocean and enriched in the regions with moderate temperature and low phosphate concentration, according to the analysis of environmental meta-omics data set. The findings of this study indicate that 2-AEP can be an alternative P source for P. tricornutum, and the utilization of 2-AEP might play an important role in environmental adaption. The ecological implications of this proposed mechanism of diatoms require further rigorous experimental verification. Overall, this study unveils a new mechanism and provides the insights for future endeavors in exploring the utilization of phosphonate by eukaryotic phytoplankton.

## MATERIALS AND METHODS

### Culture conditions and experimental setup.

In this study, progressive experiments were conducted in 3 batches to address specific issues (culture conditions provided in Table 1). Before the experiment, P. tricornutum seed culture was subjected to P starvation to deplete the intracellular P storage for 8 to 10 days, while the DIP concentration in the medium was below the detection limit (~0.3 μM). The first batch culture was aimed to investigate bioavailability of different phosphonate compounds for P. tricornutum. The second batch culture was designed to explore the 2-AEP uptake efficiency of P. tricornutum. The third batch culture was set up to determine the phospholipids under different P groups. Different P treatment groups in each batch were triplicated.

### Determination of physiological parameters (cell density, Fv/Fm, and APA).

Cell density and Fv/Fm were measured daily while the alkaline phosphatase activity (APA) was determined every other day, during the course of the experiments. The cell density was monitored using CytoFLEX flow cytometer (Beckman Coulter). Cell suspension (1 mL) sampled in a centrifuge tube was subjected to a total of 10,000 counting events for each measurement. The evaluation of cell density was determined by gating areas in the Chlorophyll A vs SSC-A dot plot in which all cells appear. The photochemical efficiency of photosystem II Fv/Fm was measured daily using a FIRe Fluorometer System (Satlantic) as described earlier (70). As a biomarker for P-stress, APA was determined by colorimetric method using p-nitrophenyl phosphate (PNPP) (Sigma-Aldrich) as the substrate according to the method described by Baker et al., 2008 (71). First, 1 mL sample was collected and 50 μL PNPP was added. After 2 h reaction, the absorbance of supernatant was measured at 405 nm. Different concentrations of p-nitrophenol (PNP) were used to draw a calibration curve, and the APA was calculated as PNP concentration per cell per hour.

### Determination of P concentration, cellular carbon, and nitrogen contents.

Cellular carbon and nitrogen contents were determined using a vario EL cube analyzer (Elementar Analysensysteme GmbH) (72). Briefly, 5 mL cell culture was collected every other day and filtered onto 25 mm GF/F glass fiber filters which had been precombusted at 450°C for 4 h. GF/F filtrates were preserved at −20°C. Before determinations, frozen filters were dried at 60°C for 8 h. After that, 500 μL of 1% HCl was dripped onto the filters and the filters were dried again at 60°C for 12 h. The carbon and nitrogen contents were calculated as per cell and C/N ratios were obtained. DIP was measured daily to monitor the scarcity of phosphate in P-depleted and phosphonate groups using molybdenum blue method (73). DOP measurement was carried out every other day during the course of the experiments. A suitable high performance liquid chromatography (HPLC) based method was established for the determination of 3 phosphonates (GLY, GLU, and 2-AEP) in salt matrix (74). Briefly, the medium filtrate was first prepared and diluted to a proper salinity and concentration. Then, a derivatizing reagent 9-fluorenylmethyl chloroformate (FMOC-Cl) with concentration of 6.0 mmol/L was used to react with the phosphonates in the diluted filtrate. After 30 min of reaction, the derivatives were determined by HPLC with fluorescent detection (excitation wavelength at 265 nm and emission wavelength at 315 nm).

### RNA-seq analysis and quantitative reverse transcription-PCR.

For each sample, 10^8^ cells were collected by centrifugation at 6000 rpm for 10 min under 4°C, then resuspended in 1 mL TRIzol reagent (Invitrogen) and stored at −80°C before RNA extraction. For RNA isolation, a Direct-zol RNA Miniprep Kit (Zymo researchwas used). The extracted RNA was dissolved in RNase-free water and stored at −80°C for subsequent high-throughput sequencing.

In total, 24 cDNA samples representing different cell growth states under different P nutrient conditions (Table S1a) were subjected to MGISEQ-2000 (2 × 150 bp) sequencing (BGI). About 6.37 Gb reads were obtained per sample after trimming the adaptors, as well as removing low quality sequences and unknown reads with extremely high N bases. The reference genome of P. tricornutum (ASM15095v2) was downloaded from ftp://ftp.ensemblgenomes.org/pub/protists/release38/fasta/phaeodactylum_tricornutum/dna//Phaeodactylum_tricornutum.ASM15095v2.dna.toplevel.fa.gz. The clean reads were assembled and blasted with the reference genome using HISAT2 (v2.0.4) (75) and reference gene using Bowtie2 (v2.2.5) (76), respectively. The raw sequence reads were submitted to the SRA at NCBI under BioProject ID PRJNA764555.

The gene expression level was calculated using RSEM (v1.2.12) (77) and normalized to fragments per kilo base of transcript per million mapped reads (FPKM) values. Genes with Log2FC (fold change) ≥ 1 and Q value ≤ 0.05 were defined as significantly DEGs by using the DESeq2(v1.4.5) (78). DEGs were subsequently mapped to Gene Ontology (GO) terms to determine the gene ontology and classified into different biological pathways according to the KEGG annotation by using Phyper (https://en.wikipedia.org/wiki/Hypergeometric_distribution) based on Hypergeometric test. The significant levels of terms and pathways were corrected by Q value with a rigorous threshold (Q value ≤ 0.05) according to the method reported by Bonferroni (79).

Selected genes were subjected to quantitative reverse transcription-PCR (RT-qPCR) to verify transcriptional expression. Specific primers (Table S3) were designed based on the unigene sequence with highest fold change obtained in DEGs profile. Ribosomal protein coding gene RPS (Phatr3_J10847) was used in the first batch experiment as a reference gene to normalize the expression of the target genes as reported (80). RT-qPCR was performed on iCycle iQ Real-Time PCR Detection System using Bio-Rad iQ SYBR green Supermix Kit (Bio-Rad Laboratories). The fold change of selected genes was determined by 2^(−ΔΔCt)^ (81).

### Lipid extraction and ^31^P NMR.

Lipid was extracted according to modified Meneses and Glonek’s method (82). After centrifugation at 5000 r/min, 4°C for 10 min, cell pellets were resuspended in chloroform/methanol (2:1 vol/vol) solution with 0.88% NaCl, and homogenized by ultrasonic extraction on ice (30 min, 100 Hz). After upper layer removal, 5 mL methanol/water (1:1 vol/vol) was added to backwash the solution. Then, the mixture was centrifuged at 5000 r/min, 4°C for 10 min. Lipid fraction that settled on the upper chloroform layer was subjected to evaporation by using nitrogen blowdown. It was then redissolved in 0.5 mL CDCl_3_/CD_3_OD (2:1 vol/vol) for solution ^31^P NMR spectroscopy using a 600-MHz Bruker AVANCE DPX spectrometer and running TopSpin3.2. The conditions were as follows: 80 ppm spectral width; 2 s relaxation delay; 3888 skans; and 14 μs pulse width.

### Phylogenetic analyses and biogeographic distribution of selected genes.

Selected genes *AP-2* (Phatr3_J18142), *CLTC* (Phatr3_EG01984), *PCYT2* (Phatr3_J40163), and *EPT1* (Phatr3_J33864) that represent key players in the proposed metabolism pathway were subjected to further analysis. To examine the prevalence and phylogenetic relationship of selected target genes among diatoms and other eukaryotes, the deduced amino acid sequences from representative organisms were retrieved from NCBI and aligned using ClustalW on the MEGA X platform (83). Phylogenetic tree reconstruction was performed using Maximum likelihood and Neighbor-joining method based on the Jones-Taylor-Thornton (JTT) matrix model with 1000 bootstraps in MEGA X (83).

Nucleotide sequences of selected genes were used as queries to BLASTX against the Ocean Gene Atlas (OGA) database (84) with the cut off e-value of 1E-10. Genes annotated as diatoms were extracted and subjected to further analysis of the global distribution pattern. Abundances were computed as reads per kilobase covered per million of mapped reads (RPKM), and the distribution pie chart was calculated by using ‘percentage of total abundance per gene’. The biogeographic distribution of selected genes in diatoms was plotted in R (v.4.1.1) using scatterpie and ggplot2 packages (85). Furthermore, the abundances of selected genes and expression of surface samples (5 m depth) were further analyzed in the context of environmental parameters through a 3-dimensional scatterplot.

### Statistical analysis.

To determine whether the difference between different groups was statistically significant, T-tests were applied to calculate the *P*-value. Statistical analysis of RNA-seq data was conducted by the BGI company. Log_2_ transformed FPKM and RPKM were used to draw the heatmap and scatterplot, respectively. Additoinally, 1 was added to each FPKM and RPKM value before log_2_ transformation to facilitate calculations.

### Data availability.

The transcriptome data are publicly accessible at the NCBI (https://www.ncbi.nlm.nih.gov/) under accession number BioProject ID PRJNA764555.
