# Erratum for Shu et al., “Transcriptomic-Guided Phosphonate Utilization Analysis Unveils Evidence of Clathrin-Mediated Endocytosis and Phospholipid Synthesis in the Model Diatom, Phaeodactylum tricornutum”

**DOI:** 10.1128/msystems.01294-22

**Published:** 2023-02-06

**Authors:** Huilin Shu, Yanchun You, Hongwei Wang, Jingtian Wang, Ling Li, Jian Ma, Xin Lin

## ERRATUM

Volume 7, issue 6, e00563-22, 2022, https://doi.org/10.1128/mSystems.00563-22. Page 9, Fig. 4a: The classification of Aureococcus anophagefferens should be Pelagophyceae instead of Phaeophyta. The corrected panel is shown below.[Fig fig1]

Figure S4b in the supplemental material: The classification of *Aureococcus anophagefferens* should be Pelagophyceae instead of Phaeophyta. The corrected figure is below.

**Figure fig1:**
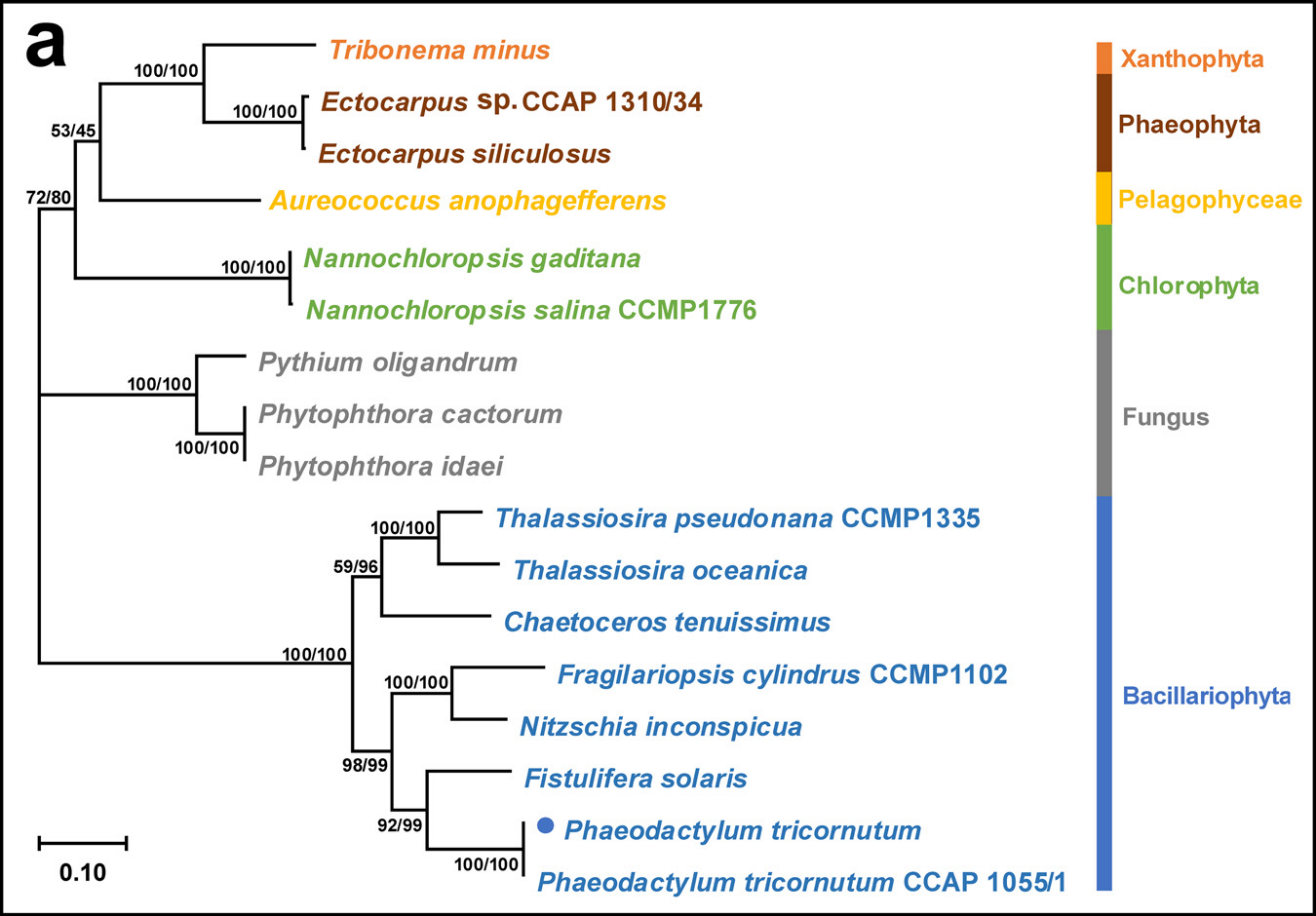


10.1128/msystems.01294-22.1 FIG S4 Corrected phylogenetic trees. Download FIG S4, TIF file, 4 MB. Copyright © 2023 Shu et al. 2023 Shu et al. https://creativecommons.org/licenses/by/4.0/ This content is distributed under the terms of the Creative Commons Attribution 4.0 International license.

