# Supplementary figures and images for: Transcriptomic-Guided Phosphonate Utilization Analysis Unveils Evidence of Clathrin-Mediated Endocytosis and Phospholipid Synthesis in the Model Diatom, Phaeodactylum tricornutum
Source: mSystems. 2022 Nov 1;7(6):e00563-22. doi: 10.1128/msystems.00563-22 (PMC9765203; doi:10.1128/msystems.00563-22)

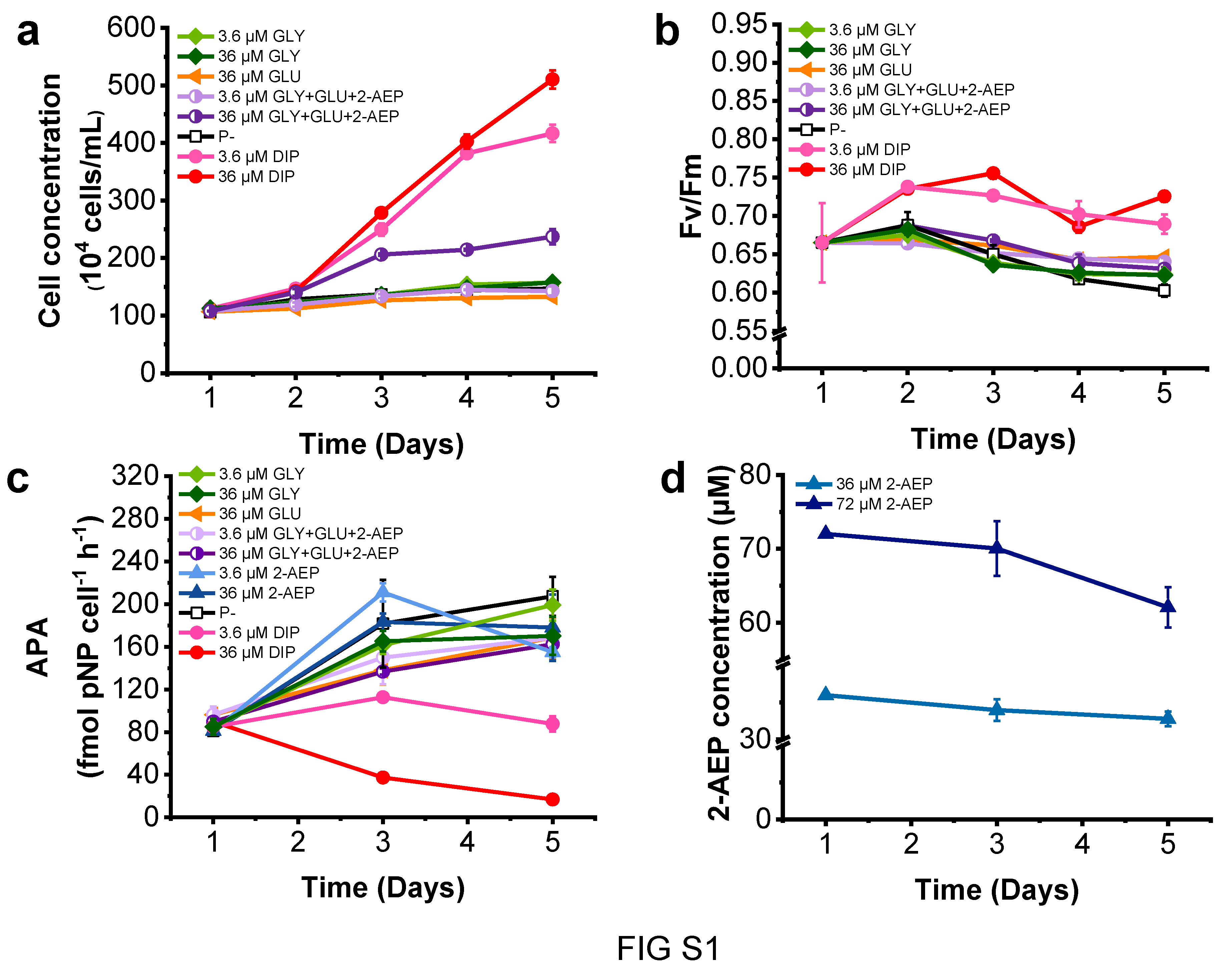

Supplement: FIG S1 [file msystems.00563-22-s0001.tif]

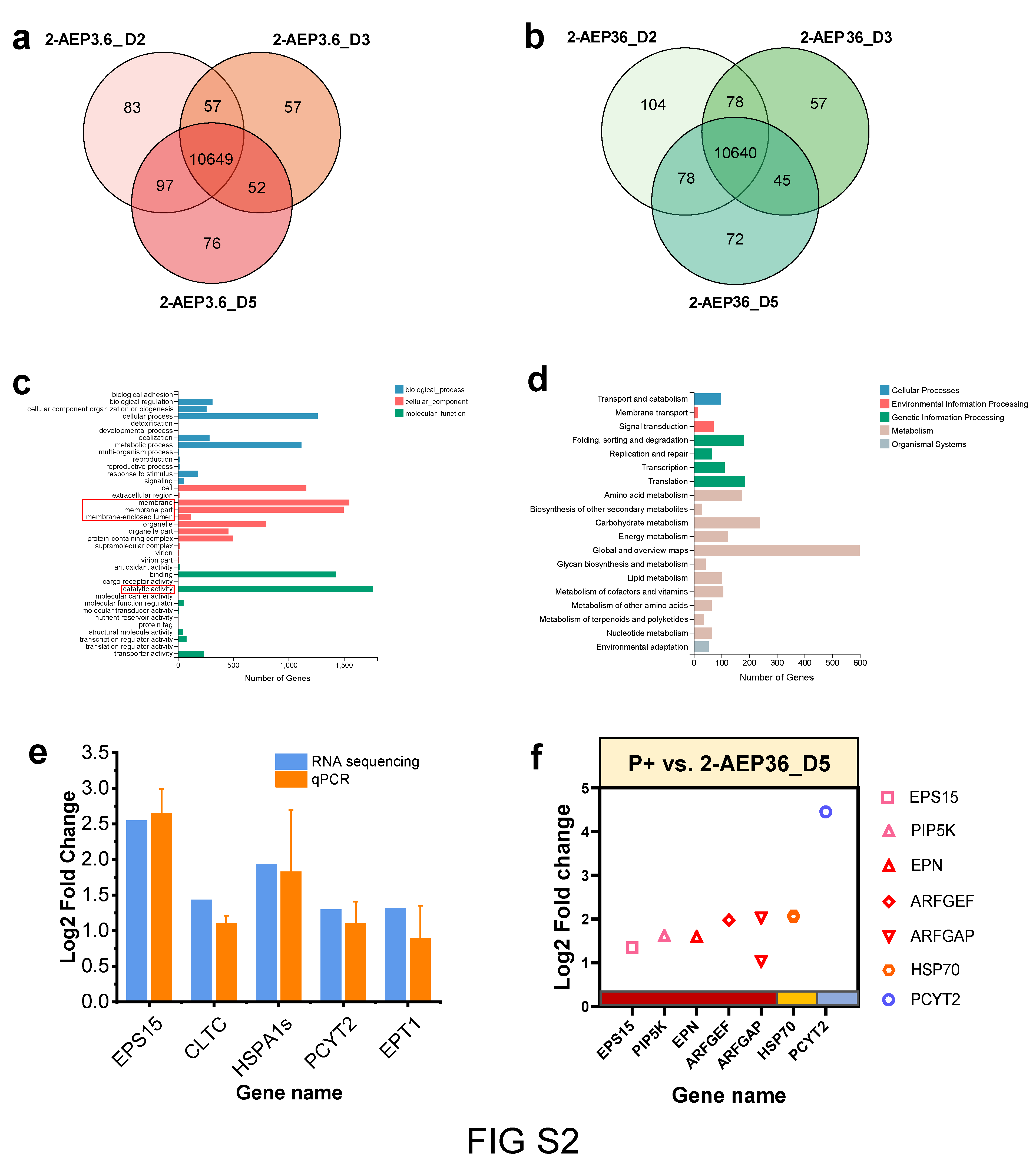

Supplement: FIG S2 [file msystems.00563-22-s0002.tif]

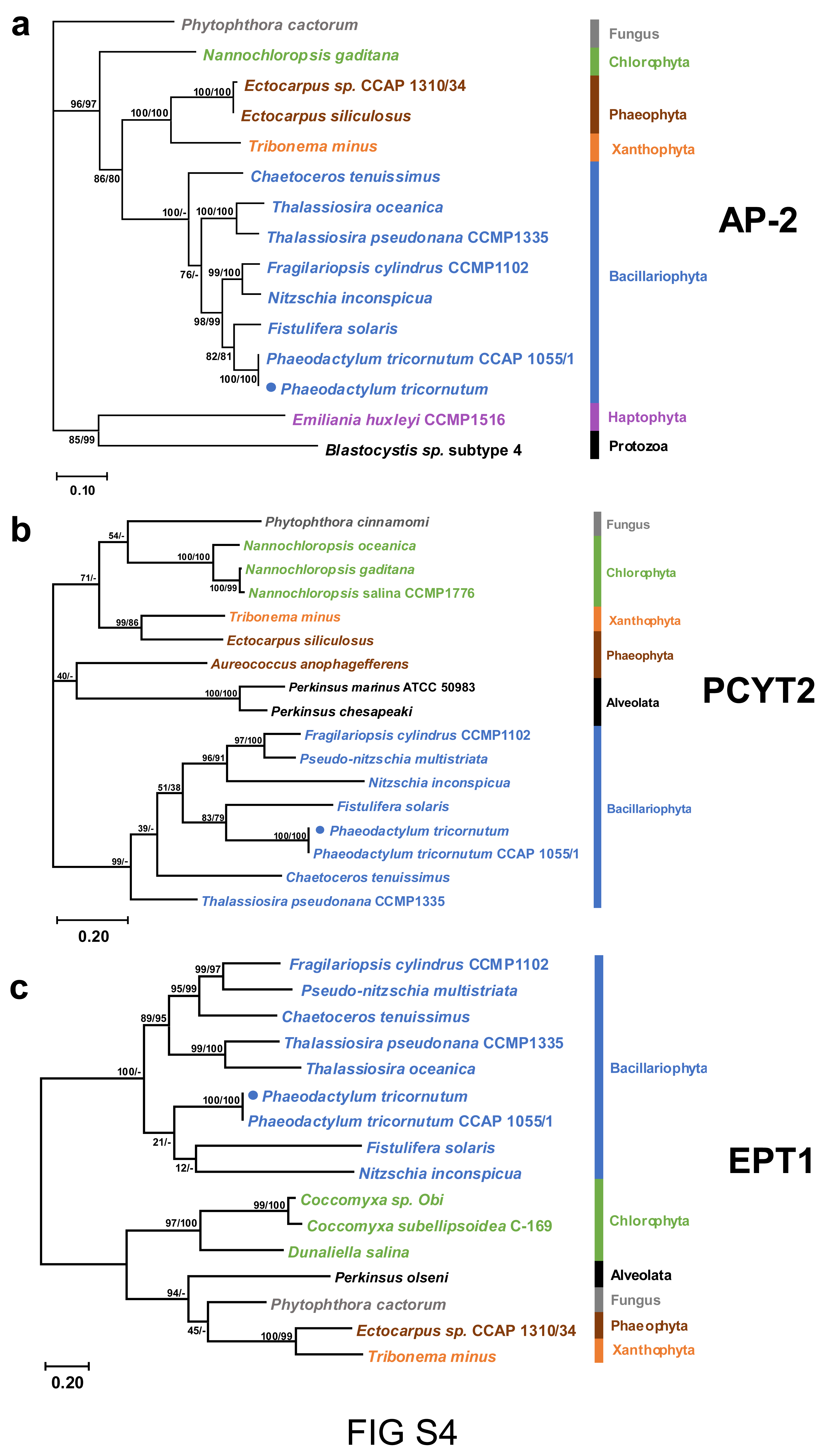

Supplement: FIG S4 [file msystems.00563-22-s0004.tif]
