# Supplementary material for: Transcriptomic-Guided Phosphonate Utilization Analysis Unveils Evidence of Clathrin-Mediated Endocytosis and Phospholipid Synthesis in the Model Diatom, Phaeodactylum tricornutum
Source: mSystems. 2022 Nov 1;7(6):e00563-22. doi: 10.1128/msystems.00563-22 (PMC9765203; doi:10.1128/msystems.00563-22)
Supplement: TABLE S2 [file msystems.00563-22-s0006.docx]

| **Gene ID** | **Gene name** | **Log2FC^a^** | **Log2FC^b^** |
| --- | --- | --- | --- |
| Phatr3_J47869 | PhoA | 4.45 (P+ vs. P-) | 6.84 (Pi_depleted_D4) |
|  |  | 1.94 (P- vs. 2-AEP36_D3) |  |
| Phatr3_J45959 | PhoD | 2.49 (P+ vs. P-) | 2.09 (Pi_depleted_D4) |
|  |  | 2.57 (P+ vs AEP36_D3) |  |
| Phatr3_J40433 | NPT | 1.51 (P+ vs. P-) | 1.37 (Pi_depleted_D4) |
|  |  | 1.98 (P+ vs AEP36_D3) |  |
| Phatr3_J47667 | NPT | 2.95 (P+ vs. P-) | 3.49 (Pi_depleted_D4) |
|  |  | 2.92 (P+ vs AEP36_D3) |  |

**Table S2** Comparison of P-stress gene markers between this study and previous reports.

a: this study, b: data source link <https://www.diatomicsbase.bio.ens.psl.eu/searchPage>
