# Supplementary material for: Transcriptomic-Guided Phosphonate Utilization Analysis Unveils Evidence of Clathrin-Mediated Endocytosis and Phospholipid Synthesis in the Model Diatom, Phaeodactylum tricornutum
Source: mSystems. 2022 Nov 1;7(6):e00563-22. doi: 10.1128/msystems.00563-22 (PMC9765203; doi:10.1128/msystems.00563-22)
Supplement: TABLE S3 [file msystems.00563-22-s0007.docx]

| **Gene ID** | **Gene name** | **Primer name** | **Sequences (5’-3’)** |
| --- | --- | --- | --- |
| Phatr3_J42442 | *EPS15* | EPS15-L | ACTACGCCTCTCACTACGGT |
|  |  | EPS15-R | CTCTGCGCCTCAATTCGTTG |
| Phatr3_EG01984 | *CLTC* | CLTC-L | CCGAACTCACGTACAAGGCT |
|  |  | CLTC-R | TCATTCAGGGCCTCGTTCAC |
| Phatr3_J54019 | *HSPA1s* | HSPA1s-L | GCGAACGTTCCATGACCAAG |
|  |  | HSPA1s-R | TCGAGTCCGTTCTTGGCTTC |
| Phatr3_J40163 | *PCYT2* | PCYT2-L | CGCACCGTATGATGTCTCCA |
|  |  | PCYT2-R | TTGGCTTGGTAGGCTTCCTG |
| Phatr3_J37086 | *EPT1* | EPT1-L | GAGTTCTCCGCTAGGTCAGC |
|  |  | EPT1-R | CCACCTTGCGGTCAACAAAG |
| Phatr3_J10847 | *RPS* (Ref gene) | RPS-L | CGAAGTCAACCAGGAAACCAA |
|  |  | RPS-R | GTGCAAGAGACCGGACATACC |

**Table S3** Primers used in RT-qPCR.
