# Supplementary figures and images for: Erratum for Shu et al., “Transcriptomic-Guided Phosphonate Utilization Analysis Unveils Evidence of Clathrin-Mediated Endocytosis and Phospholipid Synthesis in the Model Diatom, Phaeodactylum tricornutum”
Source: mSystems. 2023 Feb 6;8(1):e01294-22. doi: 10.1128/msystems.01294-22 (PMC9948723; doi:10.1128/msystems.01294-22)

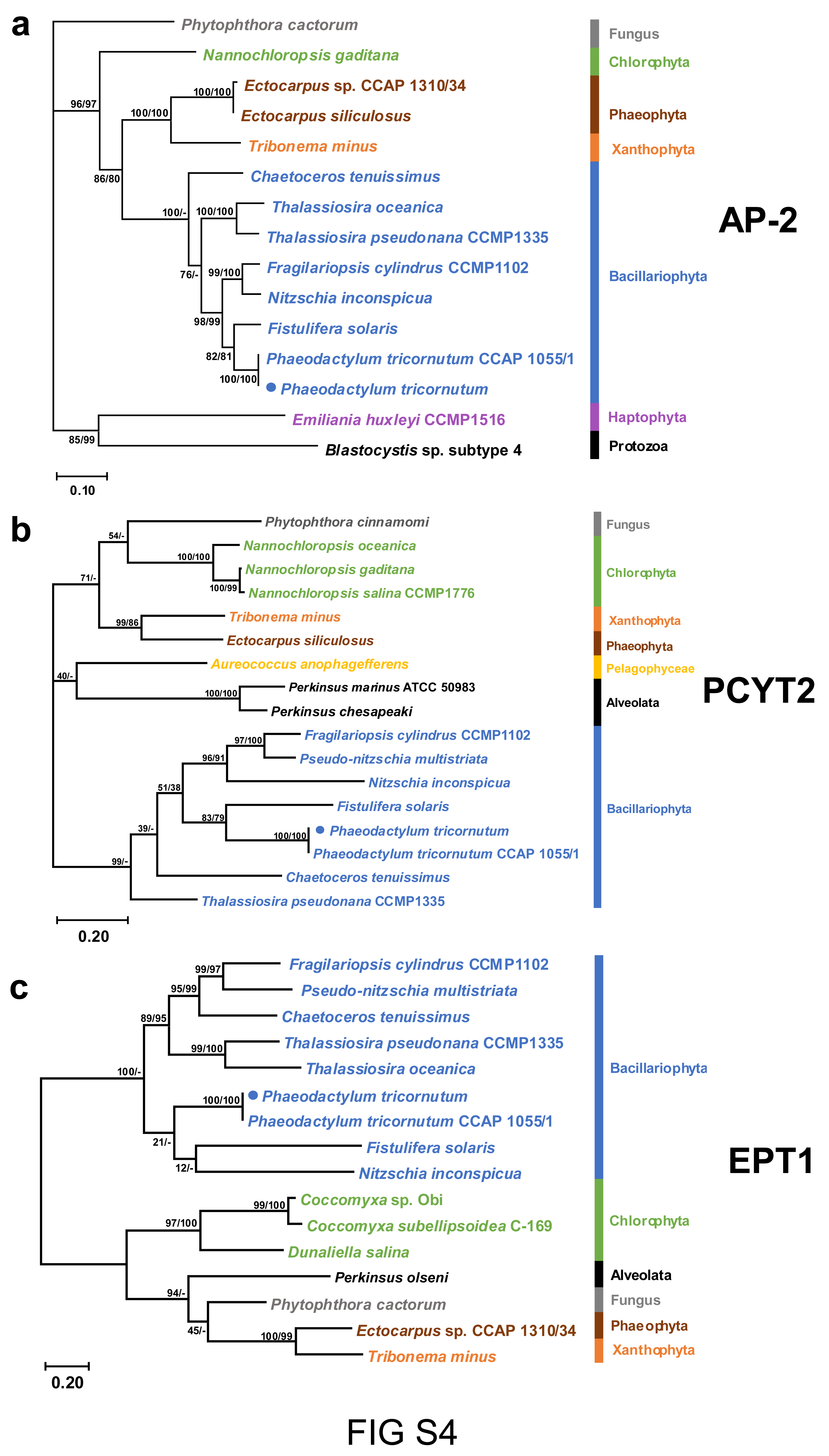

Supplement: FIG S4 [file msystems.01294-22-s0001.tif]
